# A 4-year prospective investigation of predictive effects of prepandemic sexual stigma, affective symptoms, and family support on fear of COVID-19 among lesbian, gay, and bisexual individuals

**DOI:** 10.3389/fpubh.2023.1297042

**Published:** 2024-01-08

**Authors:** Mei-Feng Huang, Yu-Ping Chang, Wen-Jiun Chou, Cheng-Fang Yen

**Affiliations:** ^1^Department of Psychiatry, Kaohsiung Medical University Hospital, Kaohsiung Medical University, Kaohsiung, Taiwan; ^2^Department of Psychiatry, School of Medicine, College of Medicine, Kaohsiung Medical University, Kaohsiung, Taiwan; ^3^School of Nursing, The State University of New York, University at Buffalo, New York, NY, United States; ^4^Department of Child and Adolescent Psychiatry, Chang Gung Memorial Hospital, Kaohsiung Medical Center, Kaohsiung, Taiwan; ^5^School of Medicine, Chang Gung University, Taoyuan, Taiwan; ^6^College of Professional Studies, National Pingtung University of Science and Technology, Pingtung, Taiwan

**Keywords:** sexual minorities, fear, COVID-19, social stigma, depression, anxiety, psychological wellbeing

## Abstract

**Aim:**

This prospective study examined whether prepandemic sexual stigma, affective symptoms, and family support can predict fear of coronavirus disease 2019 (COVID-19) among lesbian, gay, and bisexual (LGB) individuals.

**Methods:**

Data of 1,000 LGB individual on prepandemic sociodemographic characteristics, sexual stigma (familial sexual stigma [FSS] measured by the Homosexuality-Related Stigma Scale, internalized sexual stigma [ISS] measured by the Measure of Internalized Sexual Stigma for Lesbians and Gay Men, and sexual orientation microaggression [SOM] measured by the Sexual Orientation Microaggression Inventory), affective symptoms (i.e., depression measured by the Center for Epidemiologic Studies–Depression Scale and anxiety measured by the State–Trait Anxiety Inventory–State version), and family support measured by the Adaptability, Partnership, Growth, Affection, and Resolve Index were collected. Four years later, the fear of COVID-19 was assessed using the Fear of COVID-19 Scale and the associations of prepandemic sexual stigma, affective symptoms, and perceived family support on fear of COVID-19 4 years later were analyzed using multiple linear regression analysis.

**Results:**

In total, 670 (67.3%) participants agreed and completed the follow-up assessment. Greater prepandemic FSS, ISS, SOM, affective symptoms, and perceived family support were significantly associated with a greater fear of COVID-19 at follow-up.

**Conclusion:**

The identified predictors should be considered when designing interventions aimed at preventing and reducing the fear of COVID-19 in LGB individuals.

## 1 Introduction

In December 2019, the COVID-19 outbreak occurred, which spread rapidly across the globe, having major influences on human life. By 12 August 2023, more than 770 million individuals had been already infected with COVID-19 infection, and more than 6.9 million had died from it (1). The pandemic caused people worldwide to develop a fear of COVID-19 as evidenced by the results of three meta-analyses (2–4). Fear is a response pertaining to the existence of a threat and generally drives actions toward self-protection (5). Fear can lead individuals to behave in dysfunctional ways, resulting in the development of general distress and irrational beliefs (6). Several meta-analyses have concluded that the fear of COVID-19 significantly contributed to mental health disorders of individuals, including depression, anxiety, and perceived stress, and led to sleep disturbances and impaired mental wellbeing (2, 3). Research also found that the fear of COVID-19 was negatively associated with preventive behaviors (7). The results of previous studies indicated that healthcare professionals should well address the fear of COVID-19 when developing strategies to promote mental health and preventive behaviors during the COVID-19 pandemic.

The mental health and social interactions of lesbian, gay, and bisexual (LGB) individuals were deeply influenced by the COVID-19 pandemic compared with those of heterosexual individuals (8–10). The pandemic amplified existing inequities related to sexual orientation (11) and restricted the connection of LGB individuals with LGB communities, thus reducing the support that they can obtain. A meta-analysis of 15 studies investigating the mental health of LGB individuals during the pandemic revealed pooled prevalence rates of 58.6, 57.6, and 52.7% for anxiety, depression, and psychological distress, respectively (12). Therefore, fear of COVID-19 in LGB individuals warrants careful evaluation and intervention.

A review identified four categories of factors contributing to the fear of COVID-19: COVID-19 characteristics (e.g., high mortality rate and rapid transmission, variable symptomatology and disease progression, unknown origin, and lack of specific treatment models), policies for control (e.g., treatment restrictions for patients with COVID-19, quarantine, and lockdown), lack of sufficient information on the pandemic (e.g., changes in management policies, rumors about the pandemic and treatment models, and disruptions in the supply of goods and services), and contradictory statements of medical authorities and experts (13). However, no study has evaluated what prepandemic factors can predict the level of fear of COVID-19.

Perceived sexual stigma (14), emotional problems (15), and low family support (16, 17) are prevalent among LGB individuals. According to the extended parallel process model (18, 19), when individuals perceive the threat of COVID-19 and that they are at risk of being infected, they may engage in message processing both cognitively (efficacy appraisal) and emotionally (threat appraisal). If individuals believe in the effectiveness of self-protective behaviors in reducing the risk of COVID-19 infection and are confident in practicing them, they will take protective actions to avoid or mitigate the threat (i.e., danger control); otherwise, they will be too afraid to act and just try to reduce their fear (i.e., fear control) (18, 19). Several individual and environmental factors may influence LGB individuals’ cognitive and emotional appraisals of the COVID-19 threat. Experiences with sexual stigma can alter their cognitive processes, make them vigilant of their social environment, cause them to ruminate on their negative experiences, and increase their psychological distress (14). Negative emotions, such as depression, compromise individuals’ self-efficacy in managing health problems (20, 21), whereas family support enhances their self-efficacy in managing chronic illnesses (22). The Taiwanese Study of Sexual Stigma (T-SSS) conducted between August 2018 and June 2019 collected data on perceived sexual stigma (three types: familial sexual stigma [FSS], internalized sexual stigma [ISS], and sexual orientation microaggression [SOM]), family support, and affective symptoms (i.e., depression and anxiety) from 1,000 young adult LGB individuals (23–29). However, whether prepandemic perceived sexual stigma, affective symptoms, and perceived family support can predict the level of fear of COVID-19 in LGB individuals remains unclear.

This 4-year follow-up study aimed to examine the prediction of sexual stigma, affective symptoms, and family support collected before the COVID-19 pandemic on the level of fear of COVID-19 in LGB individuals. It is hypothesized that greater prepandemic FSS, ISS, SOM, and affective symptoms predicted a greater fear of COVID-19, whereas higher perceived family and peer support predicted a lower fear of COVID-19 among LGB individuals.

## 2 Methods

### 2.1 Study participants

This is a questionnaire-survey follow-up study. The T-SSS recruited a cohort of 1,000 LGB individuals (500 gay and sexual men and 500 lesbian and bisexual women) by posting online advertisements on Facebook, Twitter, LINE, and a computer bulletin board service during the period between August 2018 and June 2019 (23–29). Because the aim of the T-SSS was to evaluate the experiences of sexual stigma and mental health problems among young adult LGB individuals in Taiwan, the inclusion criteria of the T-SSS were Taiwanese LGB individuals who were 20 to 30 years of age. Those who had any form of impaired cognition (e.g., severe mental disorders, alcohol and substance intoxication to withdrawal, and cognitive impairments due to major systemic diseases) that might have interfered with the ability to understand the purpose of this study or complete the questionnaire were excluded from this study.

The present follow-up study contacted the 1,000 LGB individuals participating in the T-SSS by text messages and invited them to receive a follow-up assessment. If the contacted LGB individuals agreed to participate, a research assistant mailed them a blank informed consent form and the study questionnaire with the instructions for completing the study questionnaire. If the potential participants did not respond to the first invitation text message, the research assistant sent another text message 1 month later. A total of three invitation messages were sent to the potential participants. Those who agreed to participate in the follow-up study and sent back the written informed consent and the completed questionnaire were classified as the followed group; those who responded to none of these messages or refused participating were considered to have been lost to follow-up and were classified as the non-followed group.

### 2.2 Ethics statement

This study was approved by the Institutional Review Board of KMUH (KMUHIRB-F(I)-20210219). The participants provided their written informed consent to participate in this study. This questionnaire-survey study did not apply any experiments on humans or the use of human tissue samples. This paper conforms to the Declaration of Helsinki and Recommendations for the Conduct, Reporting, Editing, and Publication of Scholarly Work in Medical Journals.

### 2.3 Outcome variable: fear of COVID-19

Fear of COVID-19 was measured using the 7-item Fear of COVID-19 Scale (FCV-19S) (for example, “It makes me uncomfortable to think about coronavirus-19;” “My hands become clammy when I think about coronavirus-19;” “When watching news and stories about coronavirus-19 on social media, I become nervous or anxious;” and “I cannot sleep because I’m worrying about getting coronavirus-19.”) (30). Ratings were given on a 5-point scale, with a higher total FCV-19S score indicating a higher level of fear of COVID-19. Various studies have provided independent estimates of its psychometric properties obtained with samples of a generally reasonable size from diverse target populations (31). The Taiwanese version of the FCV-19S has a single-factor structure with satisfactory fit indices; the fear of COVID-19 measured by the FCV-19S was significantly associated with psychological distress measured by Depression Anxiety Stress Scale-21 among individuals in Taiwan (7). Cronbach’s alpha coefficient (α) of the FCV-19S in this study was 0.90.

### 2.4 Predicting variables at baseline

Participants’ sociodemographic characteristics, sexual stigma (FSS, ISS, and SOM), affective symptoms, and family support were measured at baseline.

#### 2.4.1 Homosexuality-related stigma scale

This study used the HRSS to measure the level of perceived FSS among LGB participants (25, 29, 32). All 12 items were rated on a 4-point scale, with a higher total HRSS score indicating a higher level of FSS (31). Cronbach’s α of the HRSS in this study was 0.93.

#### 2.4.2 Measure of internalized sexual stigma for lesbians and gay men

The traditional Chinese version of MISS-LG measures three factors of ISS, including sexuality, identity, and social discomfort (29, 33). All 17 items are rated using a 5-point scale, with a higher total MISS-LG score indicating a higher level of ISS. Psychometric evidence supports the reliability and validity of the traditional Chinese version of MISS-LG for the Taiwanese population (29). Cronbach’s α of the MISS-LG in this study was 0.76.

#### 2.4.3 Sexual orientation microaggression inventory

This study used the traditional Chinese version of SOMI (25, 34) to assess three dimensions of experienced SOM, including attitudes and expressions against, denial of, and societal disapproval of LGB sexual orientation in the previous 6 months. All 19 items are rated using a 5-point scale, with a higher total SOMI score indicating a higher level of SOM. The traditional Chinese version of the SOMI has acceptable internal consistency and concurrent validity (25). Cronbach’s α of the SOMI in this study was 0.90.

#### 2.4.4 Center for epidemiologic studies–depression scale

This study used the traditional Chinese version of the CES-D (35, 36) to measure participants’ severity of common depressive symptoms in the month before the study. All 20 items are rated on a 4-point scale, with a higher total CES-D score indicating more severe depressive symptoms. The traditional Chinese version of the CES-D has acceptable internal consistency and concurrent validity (35). Cronbach’s α of the CES-D in this study was 0.91.

#### 2.4.5 State–trait anxiety inventory–state version

The traditional Chinese version of the STAI-S was used to assess participants’ severity of current anxiety symptoms (37–39). All 20 items were rated on a 4-point Likert-type scale with scores, with a higher total STAI-S score indicating greater anxiety symptoms. The traditional Chinese version of the STAI-S has acceptable internal consistency and concurrent validity (38). Cronbach’s α of the STAI-S in this study was 0.88.

#### 2.4.6 Adaptability, partnership, growth, affection, and resolve index

The traditional Chinese version of the APGAR Index (40) was used to assess the level of perceived family support among participants. All 5 items were rated on a 4-point scale, with a higher total APGAR Index score indicating a higher family support. Cronbach’s α of the APGAR Index in this study was 0.94.

#### 2.4.7 Sociodemographic characteristics

In addition to gender (woman vs. man) and age, participants were asked “What is your highest academic qualification?” Participants were divided into two groups based on the level of education completed (college or higher vs. high school or lower). Participants were also divided into two groups based on sexual orientation (gay or lesbian vs. bisexual). Participants were asked, “Are you self-identified as a transgender?” Participants were classified into the groups of transgender or not.

### 2.5 Data analysis

All statistical analyses were conducted using SPSS 24.0 software (SPSS, Chicago, IL, United States). Depression and anxiety were transformed into affective symptoms using factor analysis. Descriptive statistics were used to summarize and analyze the participants’ sociodemographic characteristics, sexual stigma, affective symptoms, family support function, and fear of COVID-19. This study assessed whether the continuous variables were normally distributed using the definition of the absolute values of kurtosis lower than 10 and skewness lower than 3 (41). The results revealed no severe deviation.

We used several multivariate linear regression analysis models with adjustments for demographic characteristics to examine the baseline predictors in the T-SSS of the fear of COVID-19 at follow-up. In the first model with adjustment for demographic characteristics, we examined the associations of affective symptoms and family support at baseline with the fear of COVID-19 at follow-up. With adjustment for demographic characteristics, affective symptoms, and family support, FSS, ISS, and SOM at baseline were entered into the second, third, and fourth models, respectively, to examine their individual association with the fear of COVID-19 at follow-up. Because of multiple comparisons, a *p* value of <0.0125 (0.05/4) was set as significant.

## 3 Results

A total of 673 (67.3%) LGB individuals participated in the follow-up study (the followed group); 327 (32.7%) did not complete the follow-up survey (the non-followed group), including 167 responding to the invitation to follow-up but refusing to participate and 160 responding to none of the invitation messages. No significant differences in gender (χ^2^ = 0.005, *p* = 0.946), sexual orientation (χ^2^ = 2.087, *p* = 0.149), and age (*t* = 1.890, *p* = 0.059) were found between the followed and non-follow groups; however, participants in the non-follow group were more likely to have a lower education level (χ^2^ = 15.767, *p* < 0.001). Table 1 presents sociodemographic characteristics, FSS, ISS, SOM, affective symptoms, family support, and fear of COVID-19 among 637 participants. The mean and standard deviation (SD) of the FCV-19S total scores was 13.4 and 5.4, respectively.

**Table 1:** Demographics, sexual stigma, depression, anxiety, family support, and fear of COVID-19 of participants (*N* = 673).

Variable	*n* (%)	Mean (SD)	Range
Gender			
Woman	336 (49.9)		
Man	337 (50.1)		
Age at baseline (year)		24.8 (2.9)	20–30
Education level			
High school or below	55 (8.2)		
College or above	618 (91.8)		
Sexual orientation			
Bisexual	300 (44.6)		
Gay	373 (55.4)		
Transgender	19 (2.8)		
Perceived familial sexual stigma on the HRSS		26.8 (6.3)	10–40
Internalized sexual stigma on the MISS		35.6 (11.5)	17–76
Microaggression on the SOMI		42.3 (11.3)	19–78
Depression on the CES-D		18.9 (11.3)	0–57
Anxiety on the STAI		41.2 (12.7)	20–79
Family support on the Family APGAR Index		13.6 (3.6)	5–20
Fear of COVID-19 on the FCV-19S		13.4 (5.4)	7–32

APGAR Index, Adaptability, Partnership, Growth, Affection, and Resolve Index; CES-D, Center for Epidemiologic Studies Depression Scale; FCV-19S, Fear of COVID-19 Scale; HRSS, Homosexuality-Related Stigma Scale; MISS, Measure of Internalized Sexual Stigma for Lesbians and Gay Men; MoVac-COVID19S, Motors of COVID-19 Vaccination Acceptance Scale; SOMI, Sexual Orientation Microaggression Inventory; STAI, State–Trait Anxiety Inventory.

No significant differences in the levels of the fear of COVID-19 were found between participants with various genders, education levels, sexual orientations, and transgender or not (*p* > 0.05). The correlation between age and the fear of COVID-19 examined using Pearson’s correlation was non-significant (*p* > 0.05). Table 2 shows the correlations among the fear of COVID-19, FSS, ISS, SOM, affective symptoms, and family support. The correlations among all variables were statistically significant except for that between the fear of COVID-19 and family support.

**Table 2:** Correlations among the fear of COVID-19, sexual stigma, affective symptoms, and family support.

	1	2	3	4	5	6
1. Fear of COVID-19	–					
2. Familial sexual stigma	0.313**	–				
3. Internalized sexual stigma	0.193***	0.267***	–			
4. Microaggression	0.174***	0.399***	−0.186***	–		
5. Affective symptoms	0.256***	0.227***	0.276***	0.309***	–	
6. Family support	−0.012	−0.263***	−0.101**	−0.099*	−0.337***	–

*p < 0.05; **p < 0.01; ***p < 0.001.

Table 3 shows the results of the multiple linear regression analysis of the associations of baseline variables with the fear of COVID-19 at follow-up. The results of Model I indicated that after adjustment for sociodemographic characteristics, greater affective symptoms (*p* < 0.001), and family support (*p* = 0.012) were significantly associated with a greater fear of COVID-19. The results of Model II, III, and IV indicated that after adjustment for sociodemographic characteristics, affective symptoms, and family support, FSS (*p* = 0.010), ISS (*p* = 0.001), and SOM (*p* = 0.004) were significantly associated with a greater fear of COVID-19.

**Table 3:** Associations of sexual stigma, affective symptoms, and family support with fear of COVID-19: multiple linear regression analysis.

	Fear of COVID-19							
Variable	Model I		Model II		Model III		Model IV	
	B (standard error)	*p*	B (standard error)	*p*	B (standard error)	*p*	B (standard error)	*p*
Gender^a^	0.184 (0.426)	0.667	0.115 (0.426)	0.788	−0.627 (0.482)	0.194	0.064 (0.426)	0.880
Age	0.068 (0.072)	0.344	0.064 (0.072)	0.375	0.064 (0.072)	0.370	0.074 (0.072)	0.307
Education level^b^	1.286 (0.765)	0.093	1.263 (0.762)	0.098	1.112 (0.760)	0.144	1.412 (0.762)	0.064
Sexual orientation^c^	−0.332 (0.433)	0.443	−0.321 (0.432)	0.458	−0.011 (0.440)	0.980	−0.356 (0.431)	0.409
Transgender	−0.319 (1.225)	0.795	−0.212 (1.221)	0.862	−0.247 (1.215)	0.839	−0.168 (1.219)	0.891
Affective symptoms	1.588 (0.218)	<0.001	1.512 (0.219)	<0.001	1.344 (0.227)	<0.001	1.403 (0.226)	<0.001
Family support	0.148 (0.061)	0.012	0.179 (0.062)	0.004	0.139 (0.061)	0.023	0.144 (0.061)	0.018
Perceived familial sexual stigma	–	–	0.085 (0.034)	0.010	–	–	–	–
Internalized sexual stigma	–	–	–	–	0.073 (0.021)	0.001	–	–
Microaggression	–	–	–	–	–	–	0.054 (0.019)	0.004
*F*	8.292		8.115		8.900		8.358	
*p*-value	<0.001		<0.001		<0.001		<0.001	
Adjusted *R*^2^	0.071		0.078		0.086		0.081	

B, regression coefficient.

a Female as the reference.

b High school or below as the reference.

c Bisexual as the reference.

## 4 Discussion

The mean fear of COVID-19 measured by the FCV-19S among LGB individuals in this study was 13.4 (SD = 5.4). A meta-analysis found that the mean FCV-19S total score was 18.36 (SD = 5.9) among 46,223 individuals in 44 studies conducted during the period between May and July 2020 around the world (31). The present study evaluated LGB individuals’ fear of COVID-19 during the period between August 2022 and June 2023 when the COVID-19 pandemic has subsided significantly; therefore, the severity of the fear of COVID-19 was lower than that of the studies in 2020. However, a proportion of participants in this study still reported high fear of COVID-19. For example, 17.3% of participants of this study reported a total FCV-19S score of 19 or higher, higher than the mean FCV-19S score (18.36) in the 44 studies conducted in 2020 (31). Given that the fear of COVID-19 is negatively associated with mental health and protective behaviors, the fear of COVID-19 and its negative influences among LGB individuals warrant attention and evaluation. Research found that people who believe the information they receive and do not doubt it have high fear of COVID-19 during the pandemic (7). Therefore, teaching people how to distinguish between real and fake information is essential to the prevention of the fear of COVID-19. Healthcare professionals should also teach people how to respond effectively to outbreaks and avoid panic and fear. People should be taught to detect their own fear of an epidemic or pandemic and handle it appropriately.

Our results revealed that greater FSS, ISS, and SOM and higher levels of affective symptoms and family support before the COVID-19 pandemic were significantly associated with a greater fear of COVID-19 in LGB individuals. According to socioecological theory (42), individuals are embedded in the family microsystem; therefore, a family’s tolerance of sexual orientation helps young people develop a positive self-identity. By contrast, FSS causes LGB individuals to hide their sexual orientation from the family, which negatively affects their self-identity (15). Individuals with self-identity issues have difficulties in developing effective stress-coping strategies (43). SOM comprises subtle behavioral, verbal, or social indignities that express hostile, derogatory, or negative messages to LGB individuals (34, 44). Given that such SOM is omnipresent in their daily lives, they may be more vigilant in the presence of threatening clues in their environment; this explains why their fear of COVID-19 may subsequently increase. LGB individuals also faced difficulty in communicating with the SOM enactors because the enactors may view their own speech, views, and behavior as well-intentioned, mundane, or harmless (45). This might demoralize LGB individuals, compromise their self-esteem (46), and impair the development of perceived efficacy appraisals of challenges related to the COVID-19 pandemic. ISS is a process whereby individuals with LGB sexual orientation accept a public stereotyped image and transform their views on their sexual orientation (47). These individuals may anticipate social rejection, self-restrict their social activities, and be less willing to access medical care (48, 49). They may also experience limited information sources and social support during the COVID-19 pandemic, thus further increasing their fear of COVID-19.

In this study, we observed that in LGB individuals, higher prepandemic affective symptoms (i.e., depression and anxiety) significantly predicted a greater fear of COVID-19. Depression and anxiety can compromise individuals’ ability to correctly assess COVID-19 threats (e.g., causing them to amplify the threat) and their efficacy in managing the threat, thus increasing the level of the fear of COVID-19. Furthermore, prepandemic depression, anxiety, and greater fear of COVID-19 may also result from individuals’ distorted cognitive frame that contributes to catastrophic thinking, thereby increasing individuals’ difficulty in clarifying the authenticity of information and seeking help during the pandemic.

Because family members can provide information and support to help individuals manage COVID-19 threats, we hypothesized that higher prepandemic family support can predict a lower fear of COVID-19. Unlike the original hypothesis, this study found a positive association between prepandemic family support and the fear of COVID-19 at follow-up. It is possible that LGB individuals who perceived high family support may have a higher worry about family members contracting COVID-19 compared with those who perceived low family support. Meanwhile, LGB individuals who perceive high family support may have a close interaction with family members which may increase the concern about the risk of cross-infection of COVID-19 between family members and them. In spite of the positive association between perceived family support and the fear of COVID-19, family support has been demonstrated to be crucial for the mental health of LGB individuals during the COVID-19 pandemic (50, 51), and intervention programs for enhancing family support for LGB individuals are required.

As the first follow-up study examining the predictors collected before the COVID-19 pandemic for the fear of COVID-19 among LGB individuals, this study confirmed the predictive effects of prepandemic FSS, ISS, SOM, depressive and anxiety symptoms, and family support on LGB individuals’ fear of COVID-19. The results of this study further confirmed the necessity of eliminating sexual stigma and enhancing mental health for LGB individuals. In this context, antidiscrimination policies that promise protection from sexual stigma are instrumental (52). Broadening the understanding of LGB culture and raising awareness of prejudice toward GBM in family and the public constitute crucial steps to reducing the stigma surrounding non-heterosexuality (52). Studies have developed intervention programs for reducing ISS among GBM (53, 54) and SOM in the public (52, 55). Furthermore, healthcare providers should design programs aimed at enabling LGB individuals to avoid the development of mental health problems. Not only minority stress (47) but also intraminority community stress (56) cause mental health problems in LGB individuals. Helping LGB individuals develop strategies to cope with these stresses will help maintain mental health. Healthcare providers should take the influences of sexual stigma and affective symptoms into consideration when designing prevention and intervention strategies for the fear of COVID-19 among LGB individuals. For example, LGB individuals who experienced sexual stigma may have great fear of COVID-19 but be hesitant to seek medical help during COVID-19. Healthcare professionals should develop diversified access to health care, such as web-based counseling with friendly attitudes, to encourage LGB individuals to seek help in a timely manner. The mechanisms accounting for the significant association between family support and the fear of COVID-19 in LGB individuals warrant survey; if the significant association comes from the worry about family members’ contracting COVID-19 or the risk of cross-infection, it is necessary to help LGB individuals develop effective infection prevention strategies.

Several research limitations need to be noted. Because the present study collected self-reported data from LGB individuals, the results might be subject to single-rater bias. The participants were those who were interested in participating in this follow-up assessment. Moreover, this study recruited young adult LGB individuals to participate. Thus, further study is needed to examine whether the results of this study can be replicated in other groups of LGB individuals. Most (91.8%) of the participants in this study had an education level of college or above; moreover, participants in the non-follow group were more likely to have a lower education level. Whether the results of this follow-up study can be generalized to LGB individuals with a low educational level warrants further study. Furthermore, the linear regression analysis models in this study only explained a fraction of variance (<10%) in fear of COVID-19 among LGB individuals, indicating that there are factors that have not been examined in this study for their associations with the fear of COVID-19. The prediction of some prepandemic factors such as psychological characteristics (e.g., neuroticism), health status (e.g., HIV history), and economic status on the fear of COVID-19 among LGB individuals warrants further study. These variables may also serve as the third variables that may account for the associations of sexual stigma, affective symptoms, and family support with the fear of COVID-19 among LGB individuals.

## 5 Conclusion

Prepandemic FSS, ISS, SOM, affective symptoms, and perceived family support significantly predicted the severity of the fear of COVID-19 at follow-up among LGB individuals. The identified predictors, including individual and environmental factors, should be considered when designing interventions aimed at reducing the fear of COVID-19 as well as depression and anxiety among LGB individuals and modifying the general public’s attitude and prejudice toward LGB individuals.

## Data availability statement

The raw data supporting the conclusions of this article will be made available by the authors, without undue reservation.

## Ethics statement

The studies involving humans were approved by Institutional Review Board of KMUH (KMUHIRB-F(I)-20210219). The studies were conducted in accordance with the local legislation and institutional requirements. The participants provided their written informed consent to participate in this study.

## Author contributions

M-FH: Investigation, Writing – original draft. Y-PC: Resources, Writing – review & editing. W-JC: Resources, Writing – original draft. C-FY: Writing – original draft, Conceptualization, Formal analysis, Funding acquisition, Investigation, Methodology, Supervision, Validation, Writing – review & editing.
